# Supplementary material for: Analysis of influence of filling body with different filling intervals on stope stability
Source: PLoS One. 2024 Feb 1;19(2):e0293499. doi: 10.1371/journal.pone.0293499 (PMC10833552; doi:10.1371/journal.pone.0293499)
Supplement: Attachment — Submitted filename: Response_to_Reviewers.doc [file pone.0293499.s003.doc]

**《Analysis of Influence of Filling Body with Different Filling Intervals on Stope Stability》Manuscript revision comments reply**

Dear editors and experts of Plos One:

Hello! Many thanks to the editors and experts of the journal for their detailed review and pertinent opinions. Now the opinions have been revised in the paper, and detailed explanations are made here, hoping that the editors and experts can approve.I wish you good health and good luck!

**Reply to Expert Ⅲ:**

**1、It’s a mistake to write “Dear editors and experts of Journal of China University of Plos One”, this silly mistake should be avoided.**

**Reply:** Changes have been made as requested.

**2、****Line 21-22: The first letter of the first word in the Abstract should be capital..**

**Reply：**Changes have been made as requested.

The stratified backfill will lead to the reduction of the strength of the filling body, thus increasing the safety risks of the stope.

**3、One sentence is encouraged to add to the end of abstract to state the theoretical or engineering significance of the work.**

**Reply：**Relevant content has been added to the original manuscript. Add as follows:

The research results have significant reference value for improving the stope stability and selecting a reasonable interval time for stratified backfill.

**4、English should be polished throughout the manuscript.**

**Reply：**The author has polished the whole text to reduce grammatical and spelling errors.

**5、In introduction, “at home and abroad” is better replaced by “worldwide”.**

**Reply：**Changes have been made as requested.

To solve the stability problem of filling stope, numerical simulation method is widely used worldwide, which can directly reflect the change characteristics of displacement of detection points.

**6、The objective, methods** **and work procedure should be stated in the Introduction**

**Reply：**Relevant content has been added to the original manuscript. Add as follows:

In this paper, the simulation parameters are obtained after triaxial compression test by making the sample of stratified backfill. Then the FLAC 3D and MIDAS GTS NX was used to study the influence of different filling intervals on stope stability.

The objective: Therefore, this paper adopts numerical simulation method to study the influence of different filling intervals on stope stability.

Methods and work procedure: It is explained in detail in “Experimental materials and methods”

**7、Line 83: “3.3 Preliminary preparation”, “3.3” should be removed.**

**Reply：**3.3 has been removed.

**8、****Fig. 6 lacks of stress unit, N? kN? Pls also check all similar figures.**

**Reply:** When the picture was exported by software, no suitable unit was provided, so the author added the unit to the illustration. Add as follows:

Fig 6. Vertical stress nephogram of backfill stope (MPa):

Fig 9. Stope displacement diagram of filling body (mm)

**9、****Fig. 6 &9 do not illustrate the stability of neighboring stopes, only filled stope (white rectangle) is showed. It’s more important to know the stability of neighboring stopes that about to mining. These two picture should be further edited to provide more detailed information.**

**Reply:** This paper mainly studies the effect of filling interval time on stope stability. According to the simulation results, different filling intervals will affect the strength of the filling body. The shorter the filling interval, the higher the strength of the filling body, that is, the higher the stope stability, which provides a reasonable selection range for the filling interval time. The study on the stability of adjacent stope is not in the focus of this paper, so it is not included in the article.

**10、To better introduce Fig. 6 &9, stress concentration zone is encouraged to be included.**

**Reply:** The author believes that the image exported by the software can also clearly represent the stress concentration area, which may not be clear enough if it is enlarged.

**11、In Conclusion 1, the backfill suffers lower compressive stress that its ultimate stress does not mean the state of plastic zone distribution. Few backfill body is damaged by compressive stress compare to tensile stress or shear stress.**

**Reply:** Changes have been made to the expression in the text .Because the tensile stress of filling body changes little, the main contrast between compressive stress and ultimate stress, that is, the lower the compressive stress than the ultimate stress, the more stable the stope. The change of plastic zone is not considered and mentioned in this paper:

At 0h~12h, the roof tensile stress concentration area is small and the tensile stress variation is not large. The maximum compressive stress on the backfill body is less than its ultimate stress, indicating that the stope stability is stable. With the increase of filling interval, the roof tensile stress concentration area of filling stope rapidly expands and covers the mined-out area, indicating that the stope is extremely unstable.
